# Environmental and Nutritional Determinants of Macular Pigment in a Mexican Population

**DOI:** 10.1167/iovs.62.9.18

**Published:** 2021-07-09

**Authors:** Marina Green-Gomez, Rachel Moran, James Stringham, Cesar Hernández-Alcaraz, Kenny Mendoza-Herrera, J. Jans Fromow-Guerra, Alfonso Prado-Cabrero, John Nolan

**Affiliations:** 1 Nutrition Research Centre Ireland, School of Health Science, Waterford Institute of Technology, Waterford, Ireland; 2 Centro de Investigación en Nutrición y Salud, Instituto Nacional de Salud Pública, Cuernavaca, México; 3 Retina Division, Asociación Para Evitar la Ceguera en México I.A.P., México City, México

**Keywords:** macular pigment, lutein, zeaxanthin, antioxidant, nutrition

## Abstract

**Purpose:**

The carotenoids lutein (L), zeaxanthin (Z), and meso-zeaxanthin deposit at the macula as macular pigment (MP) and provide visual benefits and protection against macular diseases. The present study investigated MP, its nutritional and environmental determinants, and its constituent carotenoids in serum from a Mexican sample, in healthy participants and with metabolic diseases. Additionally, we compared these variables with an Irish sample.

**Methods:**

MP was measured in 215 subjects from a rural community in Mexico with dual-wavelength autofluorescence imaging reported as MP optical volume (MPOV). Dietary intake and serum concentrations of L and Z were evaluated.

**Results:**

The mean MPOV was 8429 (95% confidence interval, 8060–8797); range. 1171–15,976. The mean L and Z serum concentrations were 0.25 ± 0.15 µmol/L and 0.09 ± 0.04 µmol/L, respectively. The MPOV was positively correlated with L and Z serum concentrations (r = 0.347; *P* < 0.001 and r = 0.311; *P* < 0.001, respectively), but not with L + Z dietary estimates. Subjects with daily sunlight exposure of more than 50% were found to have significantly higher MPOV than those with less than 50% (*P* = 0.005). MPOV and serum concentrations of L and Z were significantly higher in the Mexican sample compared with the Irish sample, but this difference was not reflected in dietary analysis.

**Conclusions:**

These new data from a Mexican sample provide evidence of the multifactorial interactions and environmental determinants of MP such as sunlight exposure and dietary patterns. These findings will be essential for future studies in Mexico for eye health, visual function, and ocular pathology.

The macula lutea, or yellow spot, is found in the central region of the human retina. It spans approximately 5.5 mm in diameter^1^ and mediates sharp, detailed central vision.^2^ It exhibits a characteristic yellow pigmentation, called macular pigment (MP), owing to the distinctive presence of three xanthophyll carotenoids: lutein (L), zeaxanthin (Z), and meso-zeaxanthin.^3^ These xanthophyll carotenoids have several beneficial biological and optical properties, including potent antioxidant^4^^–^^6^ and anti-inflammatory activity,^7^ short wavelength (blue) light filtration,^8^ and cellular membrane stability.^9^^,^^10^ Given these properties of L, Z, and MZ, MP is thought to provide protection against oxidative stress-induced damage^11^^–^^13^ and to confer optical advantages^14^^–^^16^ to the macula. In addition, there is evidence that carotenoid supplementation decrease the risk of disease progression in patients with nonadvanced AMD,^17^ and improves visual function in both, healthy subjects^18^^–^^20^ and in patients with AMD.^21^

MP levels have been described in different populations, mainly Caucasian and Asian, in healthy participants and in patients with ocular pathologies, but not in a Mexican population. The analysis of L and Z serum concentrations and dietary intake provide a deeper understanding of MP, such as bioavailability, distribution, and potential mechanisms of uptake in the target tissue (i.e., the macula). In addition, disorders such as cardiometabolic diseases, which cause metabolic dysfunction, affect the bioavailability of L and Z, from absorption to distribution,^22^ thus affecting MP levels. Finally, certain environmental factors have been postulated to have an impact on physiologic changes, namely, sunlight exposure which affects the MP concentrations at the macula.

The primary aim of the present study was to measure MP and quantify serum concentrations and dietary intake of L and Z in a Mexican population. Additionally, we aimed to study the impact of metabolic disorders on MP in the context of a population with a high prevalence of such disorders. Finally, we compared the outcome measures of this study with a Caucasian sample from the Republic of Ireland.

## Methods

### Design and Study Population

This cross-sectional study was conducted in a rural community in Morelos, Mexico. A total of 215 participants were included between 21 and 80 years old from a primary care clinic, including both patients and family members in 2017. All subjects were informed of the aims and management of the information collected, as well as the confidentiality of the data. Consent forms and the protocol were approved by the Ethics Committee of the National Institute of Public Health, Morelos, Mexico (CI ID1444). All subjects complied fully with the tenets of the Declaration of Helsinki. Self-reported medical diagnoses included diabetes mellitus (DM), hypertension, high cholesterol, and ocular pathology (AMD, diabetic maculopathy, diabetic retinopathy, and cataracts). Participants were excluded if they had a diagnosis of a critical or acute medical condition and/or if they were taking nutritional supplementation containing L, Z, and/or MZ.

### Study Evaluations

#### Demographic, Lifestyle, Medical, and Dietary Assessment

Standardized case report forms were used to record demographics, lifestyle, medical history, and anthropometrics. Cigarette smoking was recorded by smoking status as follows: nonsmoker, if never smoked more than 100 cigarettes; former, if smoked more than 100 cigarettes in the past year and none in the last month; or current. Education was recorded as none, primary, secondary and high school, or higher (includes college degree and/or postgraduate education). Sunlight exposure was assessed by questionnaire and was based on outdoor activities and use of protective gear (i.e., hats and sunglasses). It was recorded as a percentage of sunlight exposure per day (i.e., <50%, 50%, or >50%). Physical activity was assessed as minutes per week of low to moderate activity (walking) according to the American Heart Association recommendations.^23^ Physical examination included height and body weight to calculate body mass index (BMI) as weight in kilograms divided by the square of the height in meters (kg/m^2^), categorized according to the World Health Organization classification.^24^

Dietary information was collected with a validated semiquantitative food frequency questionnaire (FFQ) used in the National Health and Nutrition Survey in Mexico.^25^ The semiquantitative FFQ recalled 139 foods over the past year before the interview and was administered by trained personnel using standardized data collection.^26^ Frequency categories for each food range from never to six times per day in the last year. Commonly used portion sizes were specified on the semiquantitative FFQ. Consumption was converted into micrograms of intake per day. Energy and nutrient values per day, including L and Z, were estimated using a food composition table compiled by the National Institute of Public Health.^26^

#### MP Measurement

MP was measured by dual-wavelength autofluorescence (AF) using the Spectralis investigational MP optical density (MPOD) module (Heidelberg Engineering GmbH, Heidelberg, Germany). Specifications and details on the technique and image acquisition have been described elsewhere.^27^ In short, pupils were dilated before MP measurement, and patient details were entered into the Heidelberg Eye Explorer (HEYEX version 1.7.1.0) software. Alignment, focus, and camera sensitivity were first optimized in near-infrared reflectance mode. Subsequently, simultaneous blue and green AF movie images were acquired, while ensuring proper alignment. The HEYEX software then averaged these images in order to generate a MP density map, where the reference eccentricity was defined at 7° retinal eccentricity from point of fixation (where the MPOD was defined as zero). A comparison of AF emission intensities generated by the RPE upon excitation by the two excitation wavelengths allows for MPOD measurements at any retinal location, via application of the Beer–Lambert law. Optical density values refer to the inverse logarithm of the amount of transmitted light relative to the total amount of incident light as it passes through some material. The MPOD is calculated as the log ratio of green-relative to blue-excited AF intensities by the RPE. Considering such mathematical computation, MPOD is a dimensionless value. In the presence of any level of MP, the intensity of the blue light-excited AF should be lower than that of green light-excited AF at the same location. Based on these fundamentals, the MPOV can be derived, whereby volume refers to the numerical integration of all MPOD values within a given area. The MPOV thus represents the sum of all MPOD values for all pixels with valid results within the area delimited by the circumference of a chosen eccentricity. Given that MPOD is dimensionless, the MPOV is, thus, unitless as well. MP measurement is reported in terms of MPOV as standardized previously.^27^

#### Carotenoid Serum Concentrations

Blood samples were collected by standard venipuncture technique in 9-mL blood collection tubes (BD Vacutainer SST Serum Separation Tubes) containing a “Z Serum Sep Clot Activator.” Collection tubes underwent thorough mixing of the clot activator. The blood samples were left for 30 minutes at room temperature to clot and then centrifuged at 725*g* for 10 minutes in a Hettich EBA 200S centrifuge (Andreas Hettich GmbH & Co. KG, Tuttlingen, Germany) to separate the serum from the whole blood. Following centrifugation, serum was transferred to light-resistant microtubes and stored at circa −80°C until the time of batch analysis. Serum carotenoid analysis was performed by high-performance liquid chromatography, using a method previously described by our laboratory.^28^ Calibration lines used, as well as lower and upper limits of quantification are as in the cited work. Serum carotenoid analysis was completed in 16 independent batches, with a maximum intraday precision of 7.28%, measured as the Residual Standard Deviation (RSD), and an interday precision of 3.16% (RSD).

#### Comparison With an Irish Sample

We conducted a comparison with MPOV, L and Z serum concentrations, and estimates of dietary intake of L and Z from an Irish sample. Data were obtained from a previously published study^29^ and were collected between 2017 and 2018. All subjects satisfied the study's regulations, complied fully with the tenets of the Declaration of Helsinki, and were granted ethical approval by local ethics committees at the Waterford Institute of Technology and South East Region. The methods and study protocols for evaluation of carotenoids in serum and tissue were identical to those used in the present study. Serum samples of both studies were analyzed in the same laboratory using the same technique, and MP was measured using the same technique and study protocol of the Spectralis investigational MPOD module (Heidelberg Engineering GmbH). Likewise, inclusion criteria were similar to the present study, participants aged 18 years and older with no critical medical conditions. A previous history of oral macular carotenoids supplementation was considered an exclusion criterion. The studied variables were the following: clinical and demographic characteristics, serum concentrations and dietary estimates of L and Z, and MPOV. Cigarette smoking, education status, and BMI are reported with the same criteria as for the Mexican sample (as described elsewhere in this article). Consumption of carotenoid-rich foods (specifically eggs, broccoli, corn, and dark leafy vegetables) was evaluated by a dietary L/Z screener offered by Tufts University to provide a scoring for carotenoid intake as an estimate of L and Z intake in micrograms per day. This method of assessing and controlling for dietary intake of carotenoids has been used with success in previously published studies.^30^^–^^33^ The reference values of L and Z used in the screener were those reported by Perry et al.^34^

### Statistical Analysis

Data were described using usual statistics, including means ± standard deviation, medians, interquartile range, minimum and maximum values for quantitative variables, and frequencies and percentages for categorical variables. To investigate the relationships between primary outcomes, Pearson's coefficient and Spearman's rank coefficient were used as appropriate for quantitative variables, and χ^2^ test for categorical variables. Data were then analyzed using multivariate analysis. Light exposure was analyzed in a regression model adjusting for protective gear (*n* = 33).

Between-group differences in the Mexican and the Irish samples were analyzed using χ^2^ tests and pairwise comparisons based on two-tailed independent samples *t*-tests. The groups differed significantly with respect to age, sex, smoking status, and BMI; hence, a multivariate regression analysis was performed to account for potential confounding factors when comparing primary outcomes. Dietary assessment methodology was different between the two groups; however, given the importance of dietary impact on tissue and serum concentrations of L and Z, both instruments were compared adjusting for confounder variables (age, sex, education, smoking, and medical diagnosis).

The statistical package IBM SPSS version 25 (Armonk, NY) was used, and a 5% significance level was applied throughout.

## Results

A total of 215 subjects were included with a mean age of 54.1 ± 11.5 years (Table 1). Eighty-one percent of the participants were women (*n* = 174), and 22% of the participants (*n* = 46) were considered healthy (Table 1).

**Table 1.** Mexican Sample: MPOV, Clinical, and Demographic Characteristics

Variable	Participants (*n* = 215)
MPOV	
Mean ± SD	8307 ± 2697
Median	8210
Range	1171–15,643
Age (y)	
Mean ± SD	54.1 ± 11.5
Range	21–80
Women, *n* (%)	174 (81.0)
Level of education, *n* (%)^*^	
None	55 (25.8)
Primary	117 (54.9)
Secondary	30 (14.1)
Higher	11 (5.2)
Smoking status, *n* (%)	
Nonsmoker	169 (79.0)
Former	25 (11.7)
Current	20 (9.3)
Medical diagnosis, *n* (%)^†^	
Healthy	46 (22)
DM	123 (57.2)
Hypertension	83 (38.8)
Hypercholesterolemia	79 (36.9)
Ocular pathology	19 (8.8)
BMI, *n* (%)^‡^	
Normal	40 (19)
Overweight	78 (37)
Obesity	93 (44)
Sunlight exposure, *n* (%)^§^^,^^‡^	
<50%	87 (41)
50% of the day	84 (40)
>50%	40 (19)
Dietary intake L + Z, µg/d	
Median	1611
Quintile 1, range	231–797
Quintile 5, range	3376–13,984
Serum L, µmol/L^‡^	
Mean ± SD	0.25 ± 0.16
Median	0.22
Min–Max	0.05–1.64
Serum Z, µmol/L^‡^	
Mean ± SD	0.09 ± 0.04
Median	0.08
Min–Max	0.03–0.24

* Data are available for 213 participants; missing data are due to failure to record during the study visit.

† Patients can be in more than one category.

‡ Data are available for 211 participants; missing data are due to failure to collect during the study visit.

§ Sunlight exposure was recorded as a percentage of sunlight exposure per day.

### MPOV Description (Mexican Sample)

The mean MPOV was 8307 (95% confidence interval [CI], 7932–8664) with minimum and maximum values of 1171 and 15,643, respectively (Table 1). Healthy subjects had a mean MPOV of 8619 (95% CI, 7795–9442). Participants diagnosed with DM, hypertension, and ocular pathology had a mean MPOV of 8339 (95% CI, 7838–8839), 8079 (95% CI, 7479–8680), and 8693 (95% CI, 7199–10,187), respectively. There were no significant differences in MPOV between healthy participants and those with any medical diagnosis including eye pathology (*P* = 0.361), even after controlling for age, sex, and BMI (*P* = 0.348).

### Linear Regression Analysis

**Figure 1.**
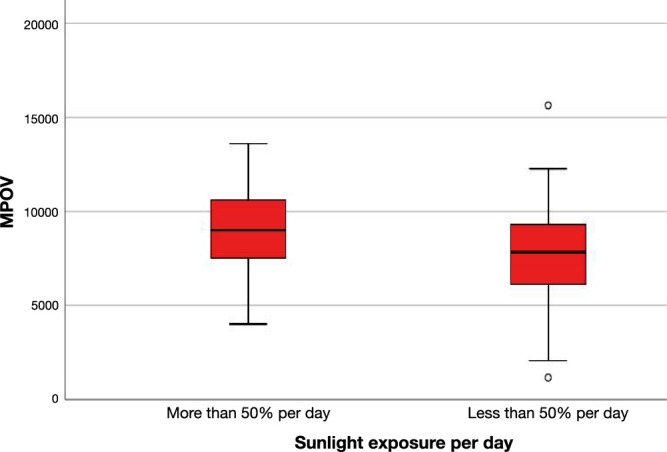
Effect of light exposure on MPOV (linear regression model, *P* = 0.007) in the Mexican sample.

Participants with longer sun exposure during the day (>50%) had higher MPOV compared with those with shorter sun exposure (<50%) (*P* = 0.005) (Fig. 1). Using a linear regression analysis, light exposure remained a positive predictor of MPOV (*P* = 0.007). The group with 50% daily exposure (*n* = 84) was not significantly different to either greater or less than 50% with respect to MPOV (*P* > 0.05 for both).

### Serum Concentrations of L and Z

**Figure 2.**
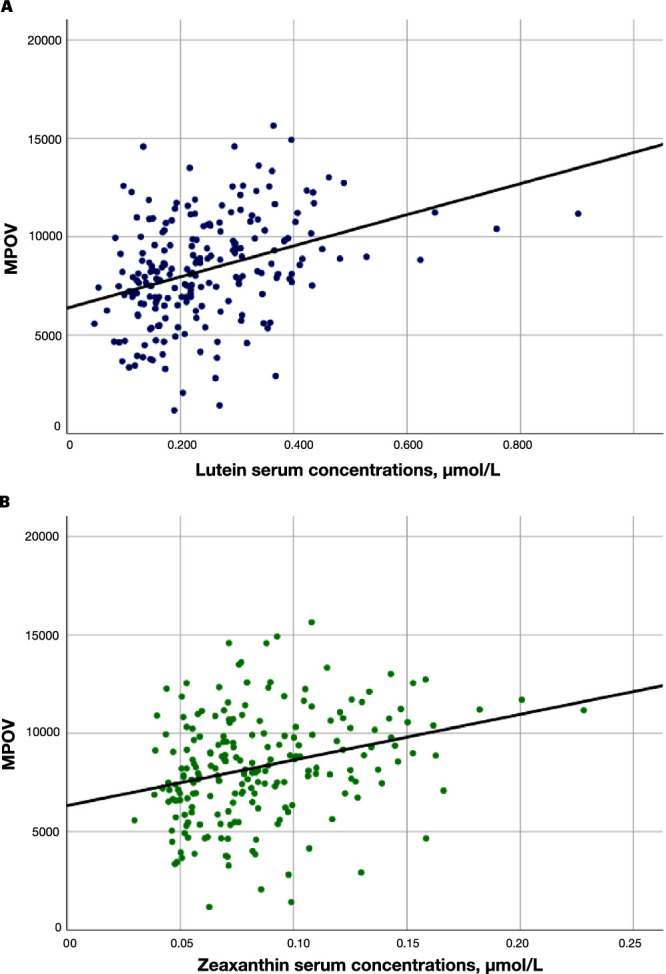
The relationship between MPOV and serum concentrations of L and Z. Linear regression analyses of MPOV and (**A**) serum L (r = 0.347; *P* < 0.001), and (**B**) Z (r = 0.311; *P* < 0.001).

The mean L and Z serum concentrations were 0.25 ± 0.16 µmol/L and 0.09 ± 0.04 µmol/L, respectively (Table 1). Serum concentrations of L and Z were positively correlated with MPOV (r = 0.347 [*P* < 0.001] and r = 0.311 [*P* < 0.001], respectively). Regression plots are shown in Figure 2. In addition, the L and Z serum concentrations were negatively correlated with age and BMI (r = − 0.149 [*P* = 0.031] and r = − 0.139 [*P* = 0.045]).

### Estimates of Dietary Intake of L and Z

**Figure 3.**
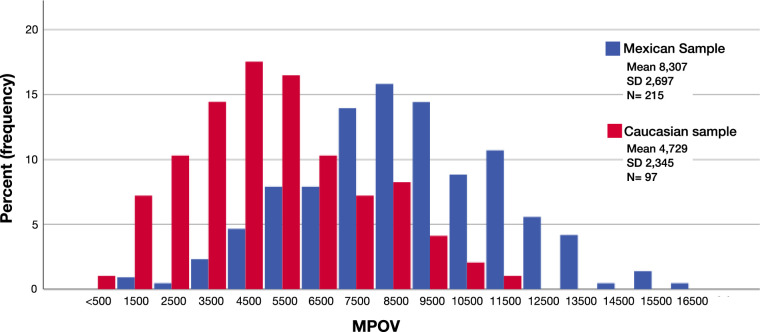
Comparison of MPOV between the Mexican and Irish samples. (Univariate analysis for multiple confounders; *P* < 0.001.)

The median L and Z dietary intake was 1611 µg/day, with a bottom quintile of 797 µg/day and a top quintile of 3376 µg/day (Table 1 and Fig. 3). There were no significant correlations between dietary intake and other variables (*P* > 0.05 for all).

### Comparison With an Irish Sample

The Mexican and Irish samples were significantly different with respect to lifestyle, health, and demographic variables (*P* < 0.001, for all) (Table 2). Therefore, these variables were adjusted in a regression model when comparing the primary outcomes.

**Table 2.** Characteristics of the Mexican and Irish Samples^*^

Variable	Mexican (*n* = 215)	Irish (*n* = 97)
Age (y), mean ± SD	54.1 ± 11.5	44.45 ± 10.2
Women, *n* (%)	174 (81.0)	48 (49.5)
BMI, *n* (%)		
Normal	40 (19)	36 (37)
Overweight	78 (37)	35 (36)
Obesity	93 (44)	26 (27)
Level of education, *n* (%)		
Primary or less	172 (81)	1 (1)
Secondary	30 (14)	38 (39)
Higher	11 (5)	58 (60)
Smoking status, *n* (%)		
Nonsmoker	169 (79.0)	51 (53)
Former	25 (11.7)	32 (33)
Current	20 (9.3)	14 (14)
Physical activity^†^ mean ± SD	147 ± 120	150 ± 205

* Variables were significantly different between samples (*P* < 0.001, for all) except for physical activity (*P* = 0.881). *P* values were based on the χ^2^ and independent-sample *t*-tests.

† Physical activity is walking in minutes per week.

**Figure 4.**
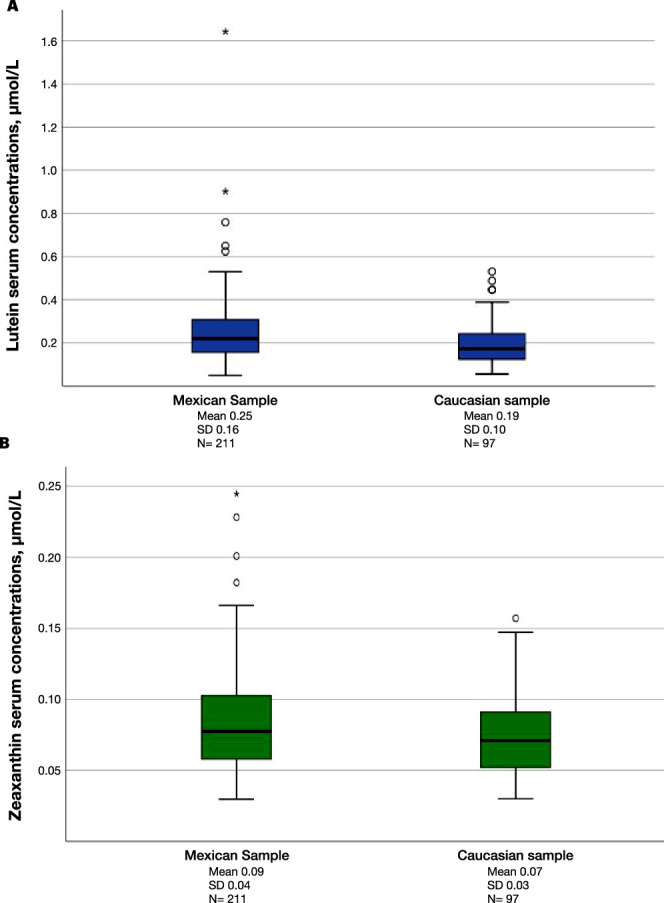
Comparison of (**A**) L and (**B**) zeaxanthin serum concentrations between the Mexican and Irish samples. The Mexican sample had higher serum concentrations of (**A**) L (*P* < 0.001) and (**B**) Z (*P* < 0.001).

**Figure 5.**
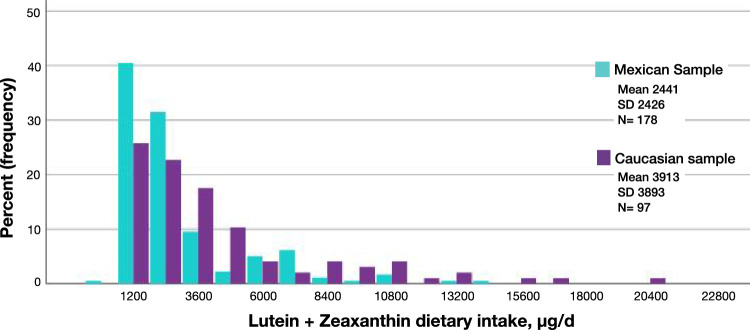
Comparison of estimates of dietary intake of L + Z between the Mexican and Irish samples (*P* < 0.001).

Table 3 presents the comparison between MPOV and its constituents in serum and diet in the Mexican and Irish samples. As shown in Figure 3 and Figure 4, the Mexican population had a significantly higher MPOV and significantly higher L and Z serum concentrations compared with the Irish sample (*P* < 0.001, for all), even after controlling for confounding variables (*P* < 0.001, for all). Interestingly, the Irish sample had higher levels of dietary intake of L + Z (*P* < 0.001) (Fig. 5).

**Table 3.** Adjusted Comparisons Between the Mexican and Irish Samples

Variable	Mexican (*n* = 215)	Irish (*n* = 97)	*P* Value
MPOV	8307 (7945–8670)	4729 (4256–5202)	<0.001
L + Z dietary Intake^*^	2441 (2081–2801)	3913 (3129–4698)	<0.001
Serum L, µmol/L	0.25 (0.23–0.27)	0.19 (0.17–0.21)	0.001
Serum Z, µmol/L	0.086 (0.08–0.09)	0.074 (0.06–0.08)	0.005

Values are means with 95% CIs.

Linear regression analyses adjusted for age, sex, BMI, education, and smoking status.

* L + Z dietary Intake is reported as micrograms per day.

## Discussion

The present study characterized MP and its determinants in a Mexican population. To our knowledge, this study is the first to evaluate MP in a Mexican sample. We showed that MPOV was positively correlated with serum concentrations of its constituent carotenoids, comparable with previous reports for different populations.^35^^–^^37^ However, there were no relationships or correlates identified between other known predictors of MP, such as cigarette smoking, BMI, or dietary intake of carotenoids, which is not consistent with previous reports.^36^^,^^38^^,^^39^

An important finding of the present study was the high concentrations of L and Z in serum and high MP values identified in this Mexican sample. For a direct comparison, we analyzed MPOV and serum carotenoid concentrations in an Irish sample from a previously published study.^29^ As shown in Figure 3 and Figure 4, the Mexican sample had significantly higher serum concentrations of L and Z by 129% and 115%, respectively, which presumably contributed to the 176% higher MPOV in the Mexican sample compared with the Irish sample.

Remarkably, and contradictory to the aforementioned findings, dietary intake of L + Z in the Mexican sample was significantly lower compared with the Irish sample (Fig. 5). Population variability is known to be largely explained by ethnic^40^ and genetic differences,^41^^–^^43^ especially when comparing a Mexican with an Irish sample, where genetics and ethnicity are considerably different. According to the last census in Ireland, the largest ethnic group was “White Irish” (82.2%)^44^ of European ancestry. Although Mexico has also European ancestry in a mixture with Indigenous ancestry, Mexicans have a multiethnicity group,^45^ generally known as Latino or Hispanic. Both populations are ethnically different, where culture, environment, and lifestyle are key contributing factors.^39^ Johnson et al.^46^ reported in a large population study of 8525 participants that the dietary intake of L and Z varies widely between individuals, across age groups, sex, and ethnicities. There are other factors that may explain this result in addition to genetics and ethnicity. First, the dietary assessment was conducted with two different instruments. The FFQ conducted in the Mexican sample was validated to evaluate the diet of Mexican adults,^26^ whereas the dietary assessment applied to the Irish sample was a screener purposely designed to identify L + Z dietary sources validated in a Caucasian population by Prof. Elizabeth Johnson from Tufts University. Such a screener helps identify food sources of L + Z otherwise neglected or infrequent by generic FFQs, resulting in a higher detection of L + Z dietary intake. In fact, the estimates of our Caucasian sample compared with other populations in the literature seem also to be higher. Olmedilla-Alonso et al.^47^ reported in a group of healthy Spanish volunteers a mean L + Z dietary intake of 1168 ± 1700 µg/d; an Australian study reported quintile 1 and 5 median of 388 µg/d and 1517 µg/d, respectively^48^; and a previous study in the Republic of Ireland reported a median of 1560 µg/d.^49^ However, despite lower dietary estimates and the methodologic limitation, to explain lower dietary intake with higher serum concentrations of L and Z in the Mexican sample compared with the Irish sample, it is important to consider the bioavailability of L and Z. The latter depends on factors such as food nutrient density, incorporation of additional lipids, dietary fibers, and thermal processing, which are among the most important.^22^^,^^50^^,^^51^ Different food matrices enhance or decrease L and Z bioavailability, which ultimately affects L and Z absorption and, hence, serum concentrations.^52^ In other words, the intake of foods with similar content of L and Z does not result in comparable serum levels. Such differences are related to the different food contributors of L + Z in both populations. In Ireland, the main sources of XCs are spinach, peas, and broccoli,^49^ whose phytomatrix decreases L and Z bioavailability,^53^ resulting in lower levels of L and Z serum concentrations. In contrast, egg and corn (as tortilla and other corn processed products) are among the most frequently consumed foods in Mexico.^46^^,^ ^54^^–^^56^ The intake of carotenoids in eggs and corn provide higher levels of bioavailable carotenoids owing to the presence of fats in the egg and the facilitated bioaccessibility of the processed matrix from the corn tortilla, respectively.^51^ Of note, it has been reported that the main source of L + Z in Mexican Americans is corn tortillas, eggs, and leafy green vegetables.^46^ In addition, the Mexican aviculture industry adds xanthophyll carotenoids, mainly L and Z, to poultry feed for commercial pigmentation of eggs.^57^ This practice results in increased concentrations of L and Z that are not accounted for in FFQs.

An interesting finding of the present study was the relationship found between sunlight exposure and MP. Sunlight exposure for a longer period on a daily basis was a significant predictor of higher MP (Fig. 2). The general finding of higher MP in a higher light intensity environment lines up with previous hypotheses about sunlight exposure and MP acting as short wavelength (blue) light protection.^58^^,^^59^ Based on our findings we hypothesize that, via an evolutionary process, perhaps similar to that of melanin accumulation in the skin according to sunlight intensity, the individuals of this sample who are exposed to significantly more sunlight on average throughout a year (2247 hours per annum in Mexico^60^) compared with the Irish sample (1424 hours per annum in Ireland^61^) have become more efficient at absorbing and depositing the xanthophyll carotenoids in the retina as a protective mechanism. Indeed, the intensity of solar radiation lessens with distance from the equator and skin pigmentation decreases, presumably to facilitate sufficient UV light interaction to produce vitamin D, and suggesting that pigmentation changes may be adaptive to manage the effects of solar radiation.^62^^,^^63^ In this regard and related to our findings, light exposure is likely to act as a stimulant to the macula to accumulate larger quantities of MP to provide higher protection.^11^^,^^64^ In other words, individuals living in an environment with a relatively high sunlight intensity would require greater antioxidant and short wave light filtering capacity to protect the retina from photo-oxidative damage, which is primarily caused by blue light.^11^^,^^58^ Moreover, it is also likely that the higher amounts of MP are essential in terms of day-to-day visual performance, which is greatly impacted by intense short wavelength light exposure,^18^^,^^65^^–^^67^ such as that produced by the sun. Previous studies suggest that exposure to UV light can degrade plasma carotenoid levels in vivo,^68^ whereas others have reported normal light exposure does not affect MPOD.^69^ However, to our knowledge, there are no data reported in the literature describing the relationship between chronic sunlight exposure and MP. To properly address this hypothesis, an interventional trial in the populations of northern and equatorial latitudes would be appropriate. In contrast, there has been an interest in investigating the relationship between sunlight exposure and AMD. Even though studies have failed to prove significance,^70^^–^^72^ it is a valuable consideration as a risk factor of AMD.^73^ On this note, if greater sunlight exposure increases the risk of developing AMD, we hypothesize that the persistent insult along with other risk factors (i.e., genetic predisposition, nutrition, smoking, aging) will progress to develop the disease, regardless of the protective mechanisms the retina has (i.e., higher MP accumulation).

Over the last two decades, research has revealed the importance of MP in human health. Intervention studies with nutritional supplements containing L and Z have shown a decreased risk of progression to severe stages in nonadvanced AMD.^17^^,^^74^^–^^76^ Other studies have shown that supplementation with L, Z, and MZ enhances visual function.^18^^,^^21^^,^^77^^–^^82^ In addition, MP has been positively correlated to brain carotenoid levels^83^^,^ ^84^ and cognitive function.^85^ On this note, the Mexican population seem to have a relatively lower prevalence of AMD; although Mexico lacks updated statistics, and it might be underestimated owing to under-reporting, studies conducted in the United States have reported that Mexican Americans have a prevalence of 3.8%^86^ to 4.0%^87^ of early AMD and 0.1%^88^ of late AMD, lower than White Americans^89^ and White Irish (6.6% and 0.6%, respectively).^90^ We propose that this finding may be due, at least in part and in addition to genes, ethnicity, and iris pigmentation, to the Mexican population having higher xanthophyll carotenoids serum and tissue concentrations.

Finally, we observed that the presence of metabolic diseases such as DM, hypertension, or hypercholesterolemia had no effect on L and Z serum concentrations and/or MPOV. Given the inflammatory nature of these conditions, it is surprising that our Mexican sample had significantly higher serum L, Z, and MPOV. However, and in line with the sunlight exposure finding, it may reflect an evolutionary and/or physiologic response of these carotenoids for protection. Previous work on MP and DM has found that those with DM have about one-half as much MP as healthy individuals.^30^ Our results may be suggestive of the importance of maintaining a diet that consistently includes L- and Z-rich foods, especially in consideration of the conditions that may lead to significant lifelong health burdens, such as DM.

The present work describes MP and its determinants for the first time in a Mexican sample and reports MP in patients with metabolic diseases. We propose that the high MP and its serum constituents observed in the Mexican sample is not only explained by ethnic and genetic differences, but by environmental determinants such as sunlight exposure and dietary patterns. It is likely that the Mexican population consumes foods with more bioavailable L and Z and/or have dietary patterns that enhance their bioavailability. On a larger scale, this adds evidence to the importance of dietary patterns in health from a serum and structural (MP) biomarker perspective.

This study was conducted in a relatively large sample of a Mexican population with standardized techniques and reproducible study protocols to enable direct comparison with an Irish sample. We report MP in terms of MPOV as has been previously validated^27^ to decrease variability in the results, specifically in an older population. Most of the participants in this sample were between 46 and 62 years old and 18% were elderly, which makes it difficult to generalize to the overall population. Nonetheless, our sample reflects the prevalence of metabolic diseases in the Mexican population^91^ and provides the first report of MP in patients with the most common metabolic diseases worldwide. One important limitation of this study is the differences between the two samples, even though they were adjusted statistically, we cannot exclude factors such as genetics and ethnicity that might remain as confounding variables. In addition, a comparison of dietary levels of L + Z was challenging not only owing to the inherent limitations of FFQs, but also to the application of different dietary survey instruments that yielded unexpectedly large L and Z intake differences between the Mexican and Irish samples. Although the cross-sectional nature of this study would never provide causality and it is a limitation on its own, it is an ideal study design to describe a sample population for the first time and provide insight to environmental and population variables, and to assess their potential interactions, which guide future prospective studies.

## Conclusions

The present study reports higher levels of MP and its serum constituents in a Mexican sample compared with an Irish sample. Nonetheless, dietary intake of L + Z appears to be lower in this Hispanic sample, which highlights the impact of dietary patterns and L and Z bioavailability on tissue concentrations (i.e., MP). Sunlight exposure is a determinant of MP and provides evidence of the relationship between MP and blue light–induced photo-oxidative damage. These new data will be essential for future studies in Mexico for eye health, visual function, and ocular pathology.
